# Identification and Characterization of N^6^-Methyladenosine CircRNAs and Methyltransferases in the Lens Epithelium Cells From Age-Related Cataract

**DOI:** 10.1167/iovs.61.10.13

**Published:** 2020-08-06

**Authors:** Pengfei Li, Hualin Yu, Guowei Zhang, Lihua Kang, Bai Qin, Yu Cao, Jiawei Luo, Xiaojuan Chen, Ying Wang, Miaomiao Qin, Jian Wu, Yemeng Huang, Xi Zou, Huaijin Guan, Yong Wang

**Affiliations:** 1 Eye Institute, Affiliated Hospital of Nantong University, Nantong, Jiangsu, China; 2 Department of Oncology, Nanjing First Hospital, Nanjing Medical University, Nanjing, Jiangsu, China; 3 Department of Ophthalmology, The Third People's Hospital of Changzhou, Jiangsu, China

**Keywords:** age-related cataract, N^6^-methyladenosine, circular RNA, ALKBH5

## Abstract

**Purpose:**

To explore the involvement of N^6^-methyladenosine (m^6^A) modification in circular RNAs (circRNAs) and relevant methyltransferases in the lesion of lens epithelium cells (LECs) under the circumstances of age-related cataract (ARC).

**Methods:**

LECs were collected from normal subjects and patients with cortical type of ARC (ARCC). M^6^A-tagged circRNAs and circRNAs expression were analyzed by m^6^A-modified RNA immunoprecipitation sequencing (m^6^A-RIP-seq) and RNA sequencing (RNA-seq). Gene Ontology (GO) annotation and Kyoto Encyclopedia of Genes and Genomes (KEGG) pathway enrichment analyses were used to predict possible functions of the m^6^A-circRNAs. Expression of m^6^A-related methyltransferase and demethytransferase was measured by quantitative real-time polymerase chain reaction. Expression and location of AlkB homolog 5 RNA demethylase (ALKBH5), a key component of m^6^A demethytransferase, were determined by Western blot and immunostaining.

**Results:**

All 4646 m^6^A peaks within circRNAs had different abundances, with 2472 enriched and 2174 subdued. The level of m^6^A abundance in total circRNAs was decreased in the LECs from ARCCs in comparison with the controls. We also found that the expression of highly m6A-tagged circRNAs was mostly decreased in comparison with non-m^6^A-tagged circRNAs. The bioinformatics analysis predicted the potential functions of m^6^A modified circRNAs and the relevant pathways that may be associated with m^6^A modified circRNAs. Among five major methyltransferases, ALKBH5 was significantly upregulated in LECs of ARCCs.

**Conclusions:**

Our data provided novel evidence regarding the involvement of circRNAs m^6^A modifications in ARC. The altered expression of methyltransferases in lens tissue might selectively change the epigenetic profile of lens genome through regulating genes that host the circRNAs, thus enhance the susceptibility to ARC. The results might provide a new insight in the molecular target of ARC pathogenesis.

Age-related cataract (ARC) is one of the leading causes of vision impairment and accounts for the majority of senile blindness worldwide.^1^ There are three main types of clinically recognized ARCs, including cortical, nuclear and sub-capsular. The cortical type is the dominant form in China.^2^ Several studies have shown that there are strong links of the cellular and molecular pathogenesis of ARC to DNA damage, DNA repair, oxidative stress, proteolysis, ubiquitination, apoptosis and autophagy.^3^^–^^7^ In particular, the repairability of DNA oxidation damage plays a crucial role in maintaining the lens’ transparency.^7^

The pathways that are essential for the ARC pathogenesis is controlled by genetic and epigenetic regulation. Our previous studies reported the shreds of evidence of non-coding RNAs in the regulation of gene expression in lens epithelium cells (LECs).^7^^−^^10^ Circular RNAs (circRNAs) are a novel class of noncoding RNAs, which are generated by the spliceosome via back splicing: the 3′ end of an exon is covalently linked to the 5′ end of an upstream exon.^11^^,^^12^ Most circRNAs originate from protein-coding genes and contain complete exons. The circRNAs can regulate the targeted genes through microRNA (miRNA) sponge or RNA-binding proteins.^13^^,^^14^ A few circRNAs affect the transcription rate of its host genes.^15^ The function of only a few circRNAs has been elucidated in cataract. For example, the circRNAs can regulate the expression of genes through miRNA in cataract.^16^^,^^17^ Circ-HIPK3 has been shown to regulate human LECs proliferation and apoptosis by circHIPK3/miR-193a/CRYAA network.^16^ The upregulation of circKMT2E may be involved in the pathogenesis of diabetic cataract by sponging miR-204-5p.^17^ However, the upstream regulatory mechanism of circRNAs modification remains unclear. Recently, a study showed that N^6^-methyladenosine (m^6^A) is a structural alteration affecting circRNA expression.^18^

M^6^A appears to be the most prevalent and functionally relevant internal modification of RNA in eukaryotic cells.^19^ The effectors in m^6^A pathways include “writers” and “erasers” that respectively install and remove the methylation.^20^ Methyltransferase-like (METTL)3, METTL14, and Wilms’ tumor 1-associating protein (WTAP) are the core components of writers.^21^ The reversible process is conducted by m^6^A erasers that include the fat mass and obesity-associated protein (FTO) and alkB homolog 5 (ALKBH5).^21^The clinical relevance of m^6^A mRNA methylation of neurological function-related genes has been reported in neurodegenerative diseases and age-related diseases.^22^ However, only a few studies have been published regarding the role of m^6^A modifications to circRNAs. One study demonstrated that consensus m^6^A motifs are enriched in circRNAs, and a single m^6^A site is sufficient to drive translation initiation.^23^ Another study showed that a different set of rules might govern m^6^A biogenesis in circRNAs compared with mRNAs because numerous m^6^A-circRNAs are generated from exons without containing m^6^A peaks in mRNAs.^24^ Even so, research on RNA methylation of circRNAs is still on the early stage, and the roles of m^6^A of circRNA in ARC pathogenesis have not been reported.

Herein, we hypothesized that m^6^A-circRNAs might be associated with the LEC lesions by regulating genes/pathways related to ARC pathogenesis. We conducted genome-wide screening of m^6^A circRNAs in the LECs from ARCCs, the cortical type of ARC. The potential functional consequences of the modification were analyzed.

## Materials and Methods

### Study Participants

This study was approved by the Ethics Committee of the Affiliated Hospital of Nantong University and carried out in accordance with the principles of the Helsinki Declaration. This study focused on the age-related cortical cataract (ARCC) because it is the major form of ARC. The patients diagnosed at III grade in the disease severity were recruited according to the Lens Opacities Classification III (LOCS III) classification.^25^ In addition, age-matched controls were included who had their transparent lens extracted due to vitreoretinal diseases. Patients with complicated cataracts due to high myopia, trauma, diabetes mellitus, uveitis, or glaucoma, and patients with systematic diseases, such as hypertension and diabetes, were excluded from the study. Following the screening criteria as mentioned above, the LECs of three controls and three ARCCs were used for the initial high throughput screening of methylated (m^6^A) RNA immunoprecipitation sequencing (MeRIP-seq), and seven controls and seven ARCCs for the follow-up quantitative real-time polymerase chain reaction (qRT-PCR) confirmation. All patient information is shown in Table 1.

**Table 1.** The Grade of Lens Opacity and Identification Codes of Controls and ARCCs

Controls				ARCCs			
Samples	Sex	Age (y)	LOCSIII	Samples	Sex	Age (y)	LOCSIII
No.1	Male	60	C0N1P0	No.1	Male	59	C3N1P1
No.2	Male	55	C0N1P0	No.2	Male	63	C3N0P1
No.3	Male	59	C0N1P0	No.3	Male	62	C3N1P1
No.4	Male	53	C0N1P1	No.4	Male	68	C3N0P1
No.5	Male	52	C0N0P1	No.5	Male	65	C4N1P1
No.6	Female	50	C0N1P0	No.6	Female	59	C3N1P1
No.7	Female	62	C0N0P1	No.7	Female	57	C3N0P1
No.8	Female	58	C0N1P0	No.8	Female	59	C3N1P1
No.9	Female	63	C0N1P1	No.9	Female	53	C4N1P1
No.10	Female	54	C0N1P0	No.10	Female	58	C3N1P1

### Cell Culture and Ultraviolet-B (UVB) Irradiation

Human lens epithelial cell line SRA01/04 was purchased from the RIKEN National Science Institute (Tokyo, Japan) and was analyzed by short tandem repeat (STR) profiling to authenticate its identity. The cells were cultured in Dulbecco's modified Eagle medium (Invitrogen-Gibco, Carlsbad, CA, USA) supplemented with 10% fetal bovine serum, 100 U/mL penicillin, and 100 U/mL streptomycin at 37°C in a humidified incubator containing 5% CO_2_. Cells were passaged at 70% to approximately 80% confluency. The cells were divided into two groups, namely as the control group and the experimental group. The cells of experimental group were exposed to UVB light. The method of UVB exposure and detailed information about UVB lamp were reported in our previous study except for the exposure time set to be 10 minutes.^26^

### Immunofluorescence

Cell samples were fixed with paraformaldehyde (Sigma-Aldrich, St. Louis, MO, USA) (4% in phosphate buffered saline solution [PBS]), blocked and permeabilized with 3% bovine serum albumin (BSA; Sigma-Aldrich) and 0.5% Triton X-100 (Sigma-Aldrich) in PBS, and incubated overnight at 4°C with the rabbit anti-ALKBH5 antibody (1:100; Abcam Ltd., Cambridge, UK) which were diluted with 1% BSA. After rewarming for one hour at room temperature, samples were incubated with the Alexa Fluor 488 labeled second antibody (1:200; Invitrogen-Gibco) for four hours at 37°C. Then, the nuclei were labeled with Hoechst (1:2000, Sigma-Aldrich). Samples were washed three times with PBS. The images were finally captured using a confocal microscope (SP8, Leica, Wetzlar, Germany).

### RNA Extraction and Quality Control

Total RNA was isolated from LECs and SRA01/04 cells by TRIzol Reagent (Invitrogen-Gibco) according to the manufacturer's instructions and as reported in our previous study.^7^ RNA concentration was determined by NanoDrop ND-1000 at 260/280 nm, and the OD260/OD280 ratio of RNA in all samples ranged from 1.8 to 2.1. Total RNA quality test was assessed by the ratio of the 18S/28S ribosomal band intensities in an ethidium bromide-containing 1% agarose gel after electrophoresis.

### MeRIP-Seq

MeRIP-seq maps m^6^A-methylated RNA. In this method, an m^6^A-specific antibody was used to immunoprecipitate RNA. Total RNAs were extracted from LECs. The RNAs were reverse-transcribed to cDNA and sequenced. Deep sequencing provided high-resolution reads of m^6^A-methylated RNA. The rRNAs were removed from the total RNA with NEBNext rRNA Depletion Kit (New England Biolabs, Inc., Ipswich, MA, USA). RNA libraries were constructed by using NEBNext Ultra II Directional RNA Library Prep Kit (New England Biolabs). M^6^A RNA-Seq service was provided by Cloudseq Biotech Inc. (Shanghai, China). Briefly, m^6^A RNA immunoprecipitation was performed with the GenSeqTM m^6^A-MeRIP Kit (GenSeq Inc., Cyberjaya, Malaysia) by following the manufacturer's instructions. Both the input samples without immunoprecipitation and the m^6^A IP samples were used for RNA-seq library generation with NEBNext Ultra II Directional RNA Library Prep Kit (New England Biolabs). The library quality was evaluated with BioAnalyzer 2100 system (Agilent Technologies, Inc., Santa Clara, CA, USA). Library sequencing was performed on an illumina Hiseq instrument with 150bp paired-end reads.

### Quantitative RT-PCR (qRT-PCR)

The mRNA levels of the m^6^A-related genes METTL3, METTL4, FTO, WTAP, and ALKBH5 were analyzed in LECs of seven controls and seven ARCCs using qRT-PCR. In brief, qRT-PCR of the transcripts of selected genes were on the basis of our previous research.^27^ Relative expression was calculated from the differences in the cycle time of an internal standard (GAPDH) compared to the target mRNA. The fold change relative to the control was determined by the comparative CT (2^-△△CT^) method. The primer pairs were designed through Primer 3.0 and blast (http://www.ncbi.nlm.nih.gov/tools/primer-blast/) to span at least one intron to avoid amplification of contaminating genomic DNA along with cDNA. The primers used in this study are presented in Table 2.

**Table 2.** Sequences of Primers Used for qRT-PCR Analysis of mRNA Levels

Name	Sequence
GAPDH	Sense: 5′CGGATTTGGTCGTATTGGG 3′
	Antisense: 5′CTGGAAGATGGTGATGGGATT 3′
METTL3	Sense: 5′TGATTGAGGTAAAGCGAGGTC 3′
	Antisense: 5′TCCTGACTGCCTTCTTGCTC 3′
METTL14	Sense: 5′AGAAACTTGCAGGGCTTCCT 3′
	Antisense: 5′TCTTCTTCATATGGCAAATTTTCTT 3′
WTAP	Sense: 5′GGCGAAGTGTCGAATGCT 3′
	Antisense: 5′CCAACTGCTGGCGTGTCT 3′
FTO	Sense: 5′TGGGTTCATCCTACAACGG 3′
	Antisense: 5′CCTCTTCAGGGCCTTCAC 3′
ALKBH5	Sense: 5′CCCGAGGGCTTCGTCAACA 3′
	Antisense: 5′CGACACCCGAATAGGCTTGA 3′

### Bioinformatics Analysis

The comprehensive function annotations of the methylation circRNAs data and circRNA-seq data were performed with Gene Ontology (GO), Kyoto Encyclopedia of Genes and Genomes (KEGG) analysis based on the DAVID 6.7 software (http://david.abcc.ncifcrf.gov/home.jsp). GO analysis was applied to predict the main functions of the target genes according to the GO project. Pathway analysis was performed to determine the significant pathways of the differential genes, according to the KEGG. CircRNA host gene analysis was established on the basis of GO predicted data for the illustration of the relationship between circRNAs and their host genes.

### Western Blot Assay

At 24 hours after UVB exposure, total protein was collected from the cells. Detail steps of Western blot assays were described in our previous article.^28^ The samples were incubated with rabbit anti-human-ALKBH5 (1:1000, Abcam) and rabbit anti-GAPDH (1:6,000, Abcam) at 4°C for 12 hours. Alkaline phosphatase-conjugated goat anti-rabbit IgG antibody (1:10,000; Santa Cruz, Dallas, TX, USA) was used as secondary antibodies.

### Statistical Analysis

Paired-end reads were harvested from Illumina HiSeq 4000 sequencer and were quality controlled by Q30. After 3′ adaptor-trimming and low-quality reads removing removal by cut adapt software (v1.9.3). First, clean reads of input libraries were aligned to the reference genome (UCSC HG19) by STAR software. Then circRNAs were identified by DCC software using the STAR alignment results. After that, clean reads of all libraries were aligned to the reference genome by Hisat2 software (v2.0.4). Methylated sites on circRNAs (peaks) were identified by MACS software. Differentially methylated sites were identified by diffReps. These peaks identified by both software overlapping with exons of circRNA were figured out and chosen by home-made scripts. GO, and Pathway enrichment analysis was performed by the source genes of differentially methylated circRNAs. All experiments were repeated three times, and all results were expressed as means ± standard deviation (SD). Statistical analysis was performed using SPSS software (Version 25.0, Armonk, NY, USA) and GraphPad Prism software 7.0 (GraphPad Software, San Diego, CA, USA). The single factor ANOVA was used for statistical analyses, and *p* values less than 0.05/0.01 were statistically significant.

## Results

### M^6^A in the Genome of LECs

**Figure 1.**
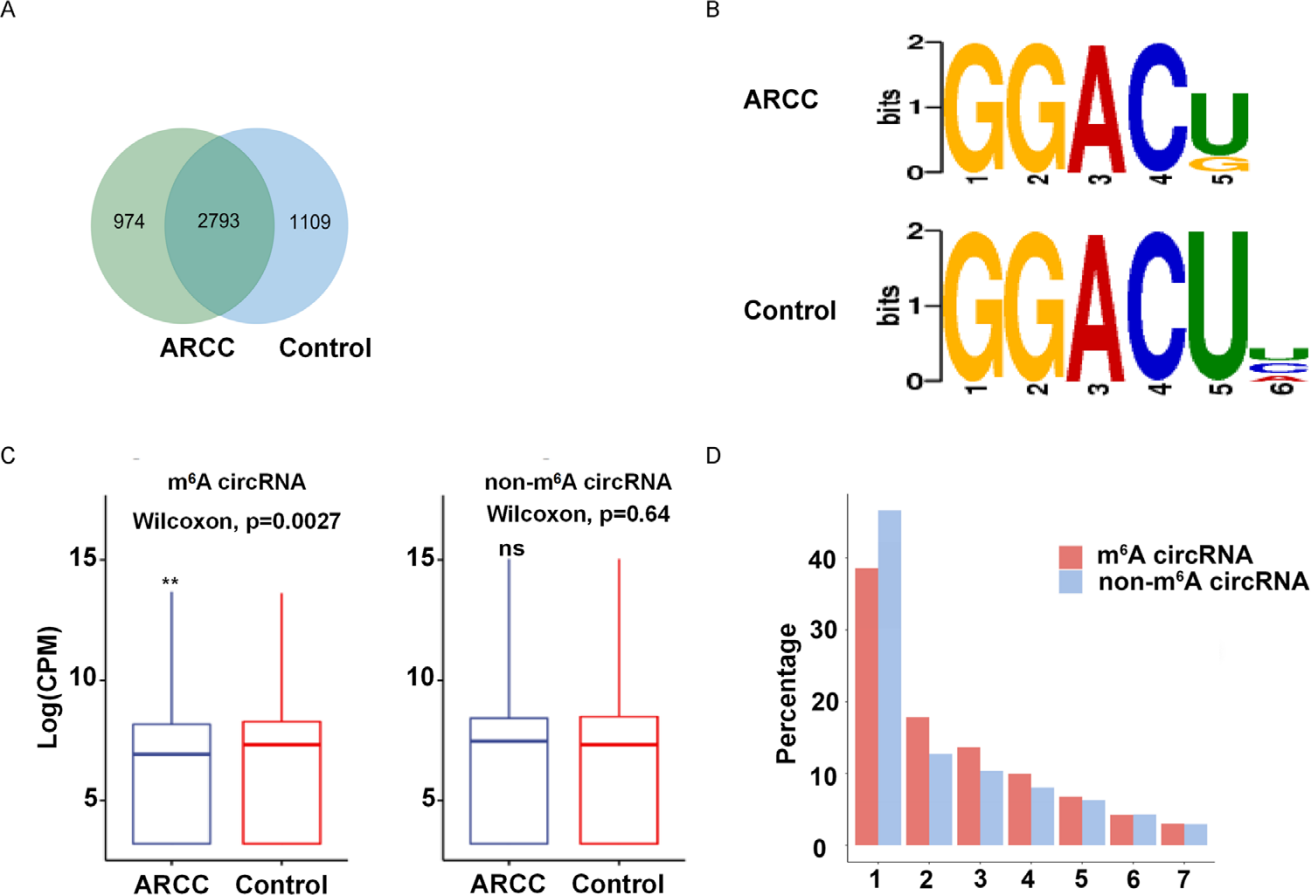
Overview of m^6^A within circRNAs in the controls and ARCCs. (**A**) Venn diagram showing the overlap of m^6^A peaks within circRNAs in two groups. (**B**) Sequence logo showing the motifs enriched across altered m^6^A-circRNAs identified come from controls and ARCCs. (**C**) The box plot shows the differential expression of m^6^A-circRNAs and non-m^6^A-circRNAs in the controls and the ARCCs. (**D**) Comparison of exon size of m^6^A-circRNAs and non-m^6^A-circRNAs.

We performed genome-wide profiling of m^6^A-modified circRNAs in LECs in three biological replicates from the controls (N = 3) and ARCCs (N = 3). The data had been submitted to gene expression omnibus (accession number, GSE153722), and we found that the m^6^A abundance in ARCC (279+974) was slightly less than controls (2793+1109). A total of 2753 m^6^A circRNAs were shared in controls and ARCCs, whereas 1109 of m^6^A-circRNAs were identified in controls but absent in ARCCs, and 974 m^6^A-circRNAs were identified in ARCCs but absent in ARCCs (Fig. 1A). A motif analysis of 2000 peaks within circRNAs with the highest scores (−10*log10, *P*-value) obtained from three biological replicates (1000 peaks per replicate) revealed consensus sequences (RRACH) in controls and ARCCs, respectively (Fig. 1B), indicating the reproducibility of the data. As shown in the left panel of Fig. 1C, the expression level of m^6^A circRNAs was lower in ARCCs than in controls. The lengths of all exons in m^6^A-circRNAs tended to be longer than those in non-m^6^A circRNAs. The majority of m^6^A circRNAs and non-m^6^A circRNAs were more commonly encoded by a single exon (Fig. 1D).

### Distribution of m^6^A Sites in LECs of Controls and ARCCs

The conjoint analysis of m^6^A-RIP-seq and RNA-seq data identified 2700 hypermethylated m^6^A peaks in circRNAs that were significantly enriched (1469; hyper-up) or subdued (1231; hyper-down), and 59 differentially circRNAs in ARCCs that were significantly upregulated (31; hypo-up) or subdued (28; hypo-down).

**Figure 2.**
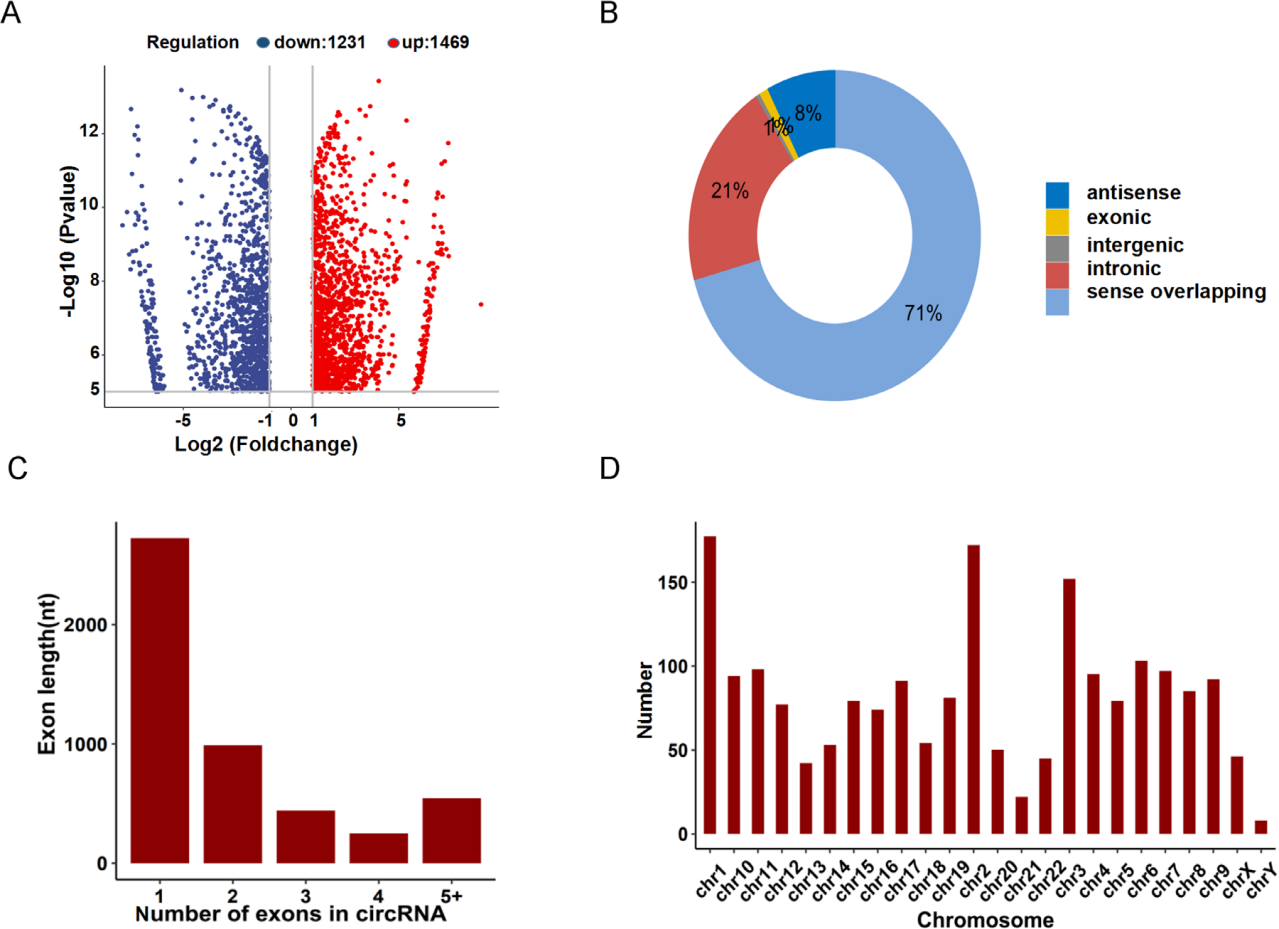
Distribution of differentially methylated N^6^-methyladenosine sites. (**A**) Volcano plots showing that the differentially m^6^A-circRNAs were expressed between in controls and ARCCs with statistical significance (fold change ≥ 1.5 and *P* < 0.05). (**B**) Genomic distribution of m^6^A circRNAs. The percentage of m^6^A-circRNAs identified under each condition is shown in parentheses. (**C**) Proportion of circRNAs harboring different number of exons by per genes. The distributions of exon length (y axis) for m^6^A circRNAs are plotted based on the number of exons spanned by each circRNA (x axis). (**D**) Chromosomal distribution of all differentially methylated sites within circRNAs.

There were 2472 m^6^A peak distributions on 1248 circRNAs with up-methylation degree, and 2174 m^6^A peaks distribution on 1148 circRNAs with down-methylation degree. Table 3 presents the top ten up and down methylated m^6^A sites within circRNAs with the highest fold change values. The volcano diagrams depict the differentially m^6^A-circRNAs that were expressed between in controls and ARCCs with statistical significance (Fig. 2A).

**Table 3.** Top 20 Differently Expressed m^6^A Peaks in ARCCs in Comparison With the Controls

Chrom	PeakStart	PeakEnd	circRNA	Foldchange	Regulation
Chr14	50325601	50326500	Chr14:50320399-50329517+	452.22	Up
Chr6	136582921	136583440	Chr6:136582168-136594325-	159.6	Up
Chr9	125902501	125902900	Chr9:125884472-125946577-	157.3	Up
Chr5	139881741	139882140	Chr5:139876200-139908465+	148.1	Up
Chr20	7984441	7984840	Chr20:7963084-7990868-	140.5	Up
Chr3	63128021	63128400	Chr3:63088368-63134477+	131.5	Up
Chr16	6746161	6746520	Chr16:6739355-6751379+	131.5	Up
Chr13	32807701	32808580	Chr13:32802721-32808868+	130.9	Up
Chr9	35309481	35309900	Chr9:35295693-35313986-	130.9	Up
Chr1	70615561	70615980	Chr1:70611392-70641665-	129.1	Up
Chr14	50325601	50326500	Chr14:50320399-50329517+	452.22	Up
Chr6	136582921	136583440	Chr6:136582168-136594325-	159.6	Up
Chr12	105423161	105423520	Chr12:105420762-105425389+	226.5	Down
Chr12	27896101	27897520	Chr12:27895922-27937336+	195.6	Down
Chr12	28671081	28671960	Chr12:28603094-28702063+	182.6	Down
Chr2	112929261	112929860	Chr2:112927341-112942737+	174.4	Down
Chr2	47673101	47673740	Chr2:47630499-47717680+	171.9	Down
Chr5	178324901	178325420	Chr5:178312615-178358327+	166.8	Down
ChrX	128223861	128224360	ChrX:128206884-128256170+	164.5	Down
Chr8	92215001	92215580	Chr8:92201750-92231233+	159.6	Down
Chr6	5409241	5409740	Chr6:5396866-5431405+	152.9	Down
Chr6	131261201	131261720	Chr6:131247745-131277633-	151	Down

We examined the distributions of genomic origins of differentially distributed m^6^A circRNAs from the eluate. Further analysis indicated that the most significantly m^6^A peaks were commonly encoded by sense overlapping sequences (Fig. 2B). Previous research indicated that most circRNAs originated from protein-coding genes that spanned two or three exons.^23^ While in our study, we identified the majority of differentially methylated circRNAs that originated specifically from protein-coding genes spanning the single exons (Fig. 2C). Furthermore, the distribution of altered m^6^A peaks in ARCCs revealed that the dysregulated m^6^A peaks were transcribed from all chromosomes, but particularly chr1, chr2, and chr3 were more prominently represented (Fig. 2D). Among this, the top three chromosomes harboring the most differentially methylated m^6^A sites were 1 (177), 2 (172), and 3 (152).

### CircRNA Profiling in LECs of Controls and ARCCs

RNAseq identified 2182 circRNAs that were shared in the controls and ARCCs, along with 4456 and 4926 circRNAs that were identified in the controls and ARCCs, respectively (Supplementary Fig. S1A). Then, we used a scatter plot to represent the up-and-down expression relationship of circRNAs (Supplementary Fig. S1B). Compared with the controls, 8794 circRNAs were observed to be differentially expressed (fold change≥1.5) in ARCCs, including 4233 upregulated and 4561 downregulated. The majority of total circRNA species that originated from protein-coding genes spanned the single exons (Supplementary Fig. S1C). Further analysis showed that the most significantly circRNAs were also encoded by sense overlapping sequences (Supplementary Fig. S1D). Meanwhile, the distribution of circRNAs in ARCCs revealed that dysregulated circRNAs were transcribed from all chromosomes, but chr1, chr2 and chr3 were dominatingly represented (Supplementary Fig. S1E).

In addition, we found that there were 2472 differently upregulated circRNAs and 1248 downregulated circRNAs in ARCCs. The top 20 altered circRNAs are listed in Table 4. GO analysis also revealed the top ten GO associated with up- or downregulated circRNAs as shown in Supplementary Fig. S2A and Supplementary Fig. S2B, respectively. The majority of differentially expressed circRNAs were upregulated.

**Table 4.** Top 20 Differently Expressed circRNAs in ARCCs

Chrom	logFC	*P* Value	Regulation	Best transcript	GeneName	Catalog
chr16:53289512- 53297009+	7.051200309	0.020452486	Up	NM_025134	CHD9	Exonic
chr13:33016525- 33018263-	7.031613681	0.021216355	Up	NM_001278432	N4BP2L2	Exonic
chr5:7036594- 7039376+	7.014825203	0.02187616	Up	ENST 00000512838	RP11-122F24.1	Sense overlapping
chr9:125941286- 125946577-	7.00506637	0.022285553	Up	NM_018387	STRBP	Exonic
chr8:99538970- 99560389-	6.987047913	0.023026542	Up	NM_006281	STK3	Exonic
chr1:94341824- 94343418-	6.960740953	0.024158725	Up	NM_014597	DNTTIP2	Exonic
chr19:18546126- 18546486-	6.958724555	0.024248228	Up	NM_016368	ISYNA1	Sense overlapping
chr9:710804- 713464+	6.951246257	0.024585349	Up	NM_015158	KANK1	Exonic
chr2:136418840- 136437894+	6.929833224	0.025546708	Up	NM_015361	R3HDM1	Exonic
chr11:130011393- 130011900+	6.922345403	0.025890608	Up	NM_001642	APLP2	Sense overlapping
chr1:51121114-5 1210447-	−7.634527865	0.006526426	Down	NM_007051	FAF1	Exonic
chr22:46189425- 46202902+	−7.489323778	0.009122695	Down	NM_013236	ATXN10	Sense overlapping
chr8:30938383- 30954366+	−7.486273726	0.009186889	Down	NM_000553	WRN	Exonic
chr5:38743226- 38744119-	−7.471430538	0.009493453	Down	NR_109951	OSMR-AS1	Sense overlapping
chr6:13579683- 13601181+	−7.451469074	0.009915108	Down	NM_012241	SIRT5	Exonic
chr7:128655033- 128658211-	−7.436341897	0.010240094	Down	NM_012470	TNPO3	Exonic
chr11:77330651- 77336860-	−7.297185761	0.01364152	Down	NM_001293	CLNS1A	Sense overlapping
chr12:122361528- 122372262+	−7.17357918	0.017305858	Down	NM_144668	WDR66	Exonic
chr3:185155235- 185165735+	−7.155758828	0.017883108	Down	NM_004721	MAP3K13	Exonic
chr13:61013822- 61068709+	−7.057641015	0.021452948	Down	NM_030794	TDRD3	Exonic

### Functional Annotation of the Distinctly Distributed m^6^A circRNAs

**Figure 3.**
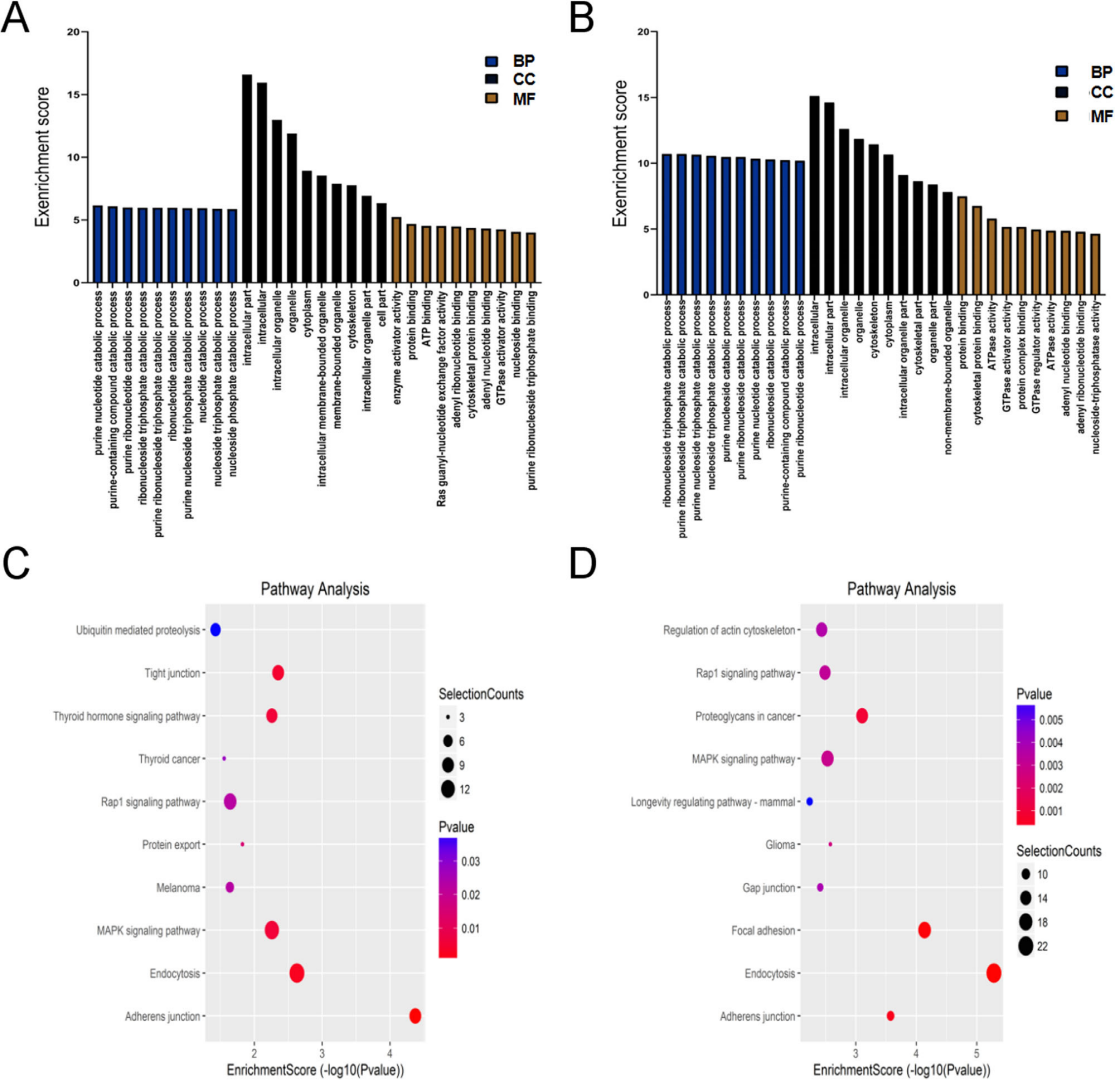
Gene ontology enrichment and pathway analysis of altered m^6^A circRNAs. (**A, B**) Major enriched and meaningful GO terms of upregulation and downregulation m^6^A peaks circRNAs. (**C, D**) The top ten significantly enriched pathways of upregulation and downregulation m^6^A peaks circRNAs.

To explore the physiological and pathological significance of m^6^A modification in ARCC, GO analysis and KEGG pathway analysis were performed for the altered m^6^A peaks. GO analysis revealed that the upregulated peaks in ARCC were significantly associated with purine ribonucleoside triphosphate catabolic process and ribonucleoside triphosphate catabolic process (ontology: biological process), intracellular and intracellular part (ontology: cellular component), and cytoskeletal protein binding (ontology: molecular function; Fig. 3A). The downregulated peaks were significantly associated with purine nucleotide catabolic process and purine-containing compound catabolic process (ontology: biological process), intracellular part (ontology: cellular component) and enzyme activator activity (ontology: molecular function; Fig. 3B). Pathway analysis showed that upregulated peaks in ARCC were significantly associated with focal adhesion and endocytosis (Fig. 3C). The downregulated peaks were significantly associated with adherens junction and endocytosis (Fig. 3D).

### M^6^A Level and Expression of circRNAs

**Figure 4.**
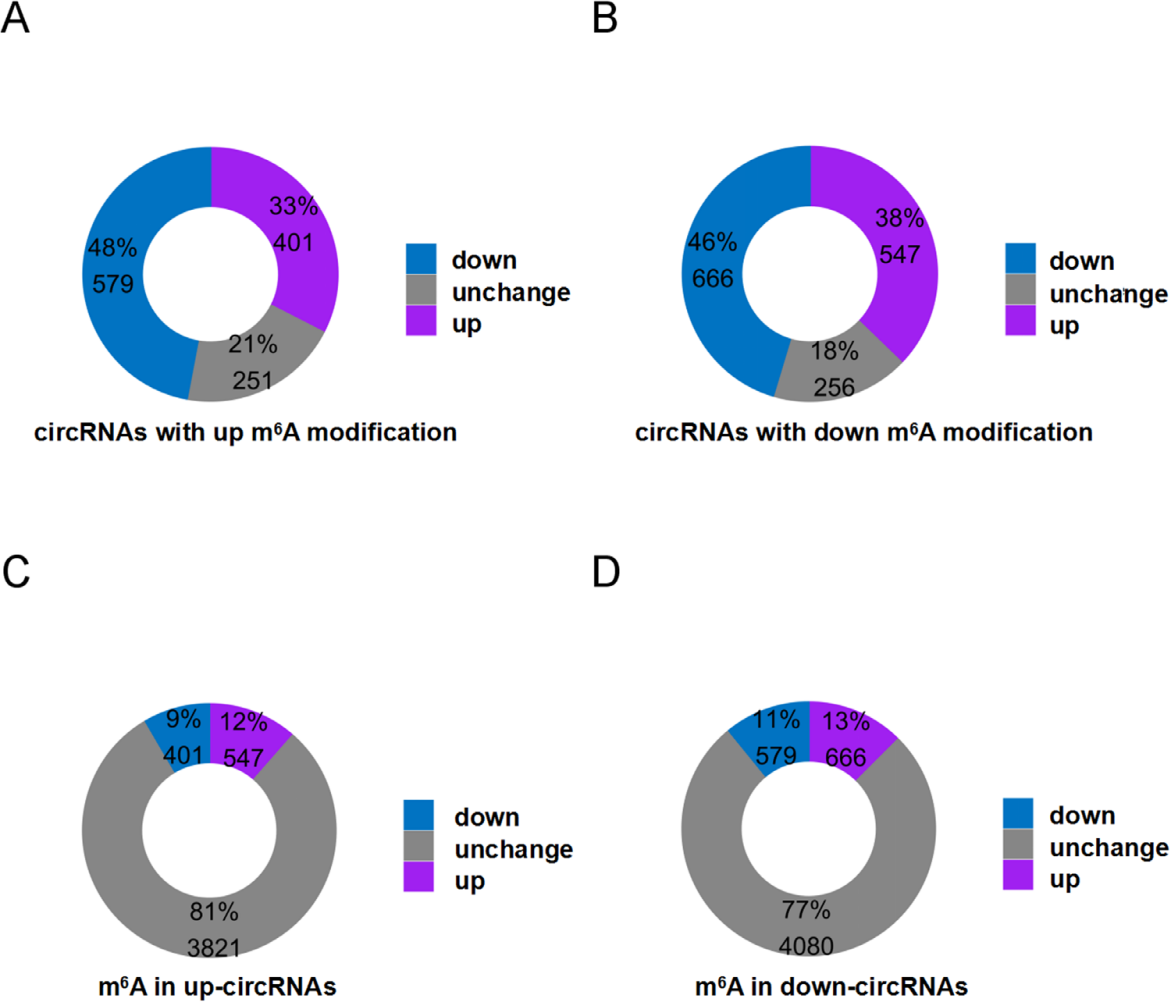
The relationship of m^6^A level and circRNAs abundance in ARC. (**A, B**) CircRNAs were more down regulated in the altered m^6^A modification of circRNAs in ARC. The percentage and number of circRNAs identified under each condition is shown in the pie chart. (**C, D**) M^6^A modification tagged circRNAs were more elevated in the upregulated and downregulated cirRNAs. The percentage and number of m^6^A-circRNAs identified under each condition is shown in the pie chart.

To explore whether m^6^A methylation would influence circRNAs expression level, the expression of the 2700 differentially m^6^A circRNAs was examined. The majority of circRNAs with abundant m^6^A were less expressed than the non-m^6^A circRNA (Figs. 4A, 4B). Whether the m^6^A modification of circRNA was upregulated or downregulated, it was found that the expression of most circRNA decreased. Moreover, expression of m^6^A circRNAs was significantly downregulated in ARCCs compared with controls, suggesting that m^6^A may downregulate the expression of circRNAs in ARCCs (Fig. 1C, *P* = 0.0027). Fewer m^6^A circRNAs (21%) were detected in upregulated circRNAs than those in downregulated circRNAs (24%) (Figs. 4C, 4D).

### Conjoint Analysis of m^6^A-RIP-seq and RNA-seq Data

**Figure 5.**
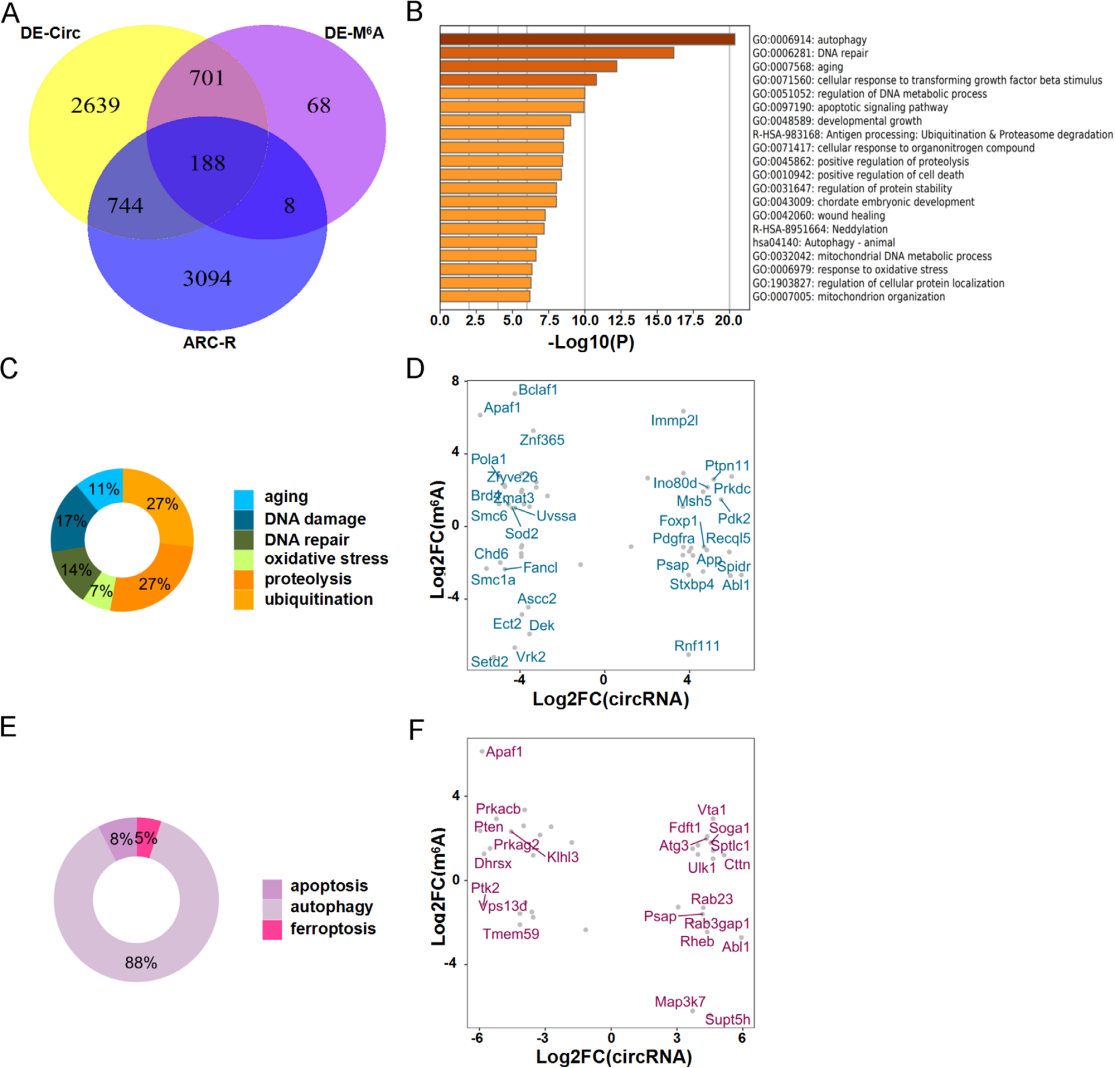
Comprehensive analysis of m^6^A-IP-seq and RNA-seq to screen for circRNA host genes associated with the pathogenesis of ARC. (**A**) Combining analysis of differentially methylated circRNAs and differentially expressed circRNAs and the intersection of host genes associated with ARC pathogenesis, which shows by using Venn diagrams. (**B**) Major enriched and meaningful GO terms of host genes associated with ARC pathogenesis. (**C**) Use pie chart to classify the results in Figure A. (**D**) The scatter plot shows the enrichment of genes associated with the ARC mechanism. (log^2^ foldchange (circRNAs) on the horizontal axis and log^2^ foldchange (m^6^A circRNAs) on the vertical axis). (**E**) Use pie chart to classify the results in Figure C. (**F**) The scatter plot shows the enrichment of genes associated with the cell death pathway. (log^2^ foldchange (circRNAs) on the horizontal axis and log^2^ foldchange (m^6^A circRNAs) on the vertical axis).

To explore the pathological significance of m^6^A modification in ARCCs, we attempted to link MeRIP-seq and RNA-seq data with genes correlated the pathogenesis of ARC. We showed the results of the screening by using the Venn diagram (Fig. 5A). In response to the oxidative damage mechanism of ARC, we screened for DNA damage, DNA repair, aging, autophagy, ferroptosis, proteolysis, and oxidative stress pathway-related circRNAs and their corresponding host genes from RNA methylation sequencing data.

GO annotation can evaluate the function enrichment, as well as gain an insight into functions of all differentially methylated circRNAs. We performed a targeted search between in MeRIP-seq data and RNA-seq data by GO analysis, using the keywords including oxidative stress, DNA repair, DNA damage, autophagy, ferroptosis, aging, apoptosis, pyroptosis, DNA repair, ubiquitination, and proteolysis (Fig. 5B). We also used the pie chart to show the proportion of each mechanism in ARC (Figs. 5C, 5D). As shown in Figures 5C and 5D, the proportions of genes related to oxidative damage/repair and autophagy rank on the top. Therefore we used a scatter plot to show that the more abundant mechanisms correspond to the host genes of differential methylation of circRNAs. In response to the oxidative damage mechanism of ARC, we not only screened DNA damage, DNA repair and oxidative stress pathway-related circular RNA from MeRIP-seq data and RNA-seq data, but also labeled the corresponding host RNAs (Fig. 5E). On the other hand, we also screened for autophagy, apoptosis, ferroptosis, and pyroptosis. We found that autophagy is highly correlated with ARC among this. Because pyroptosis did not screen out the differential circRNAs associated with it, only three of them were shown (Fig. 5F).

### The Expression of Methyltransferases in vivo and in vitro Model

**Figure 6.**
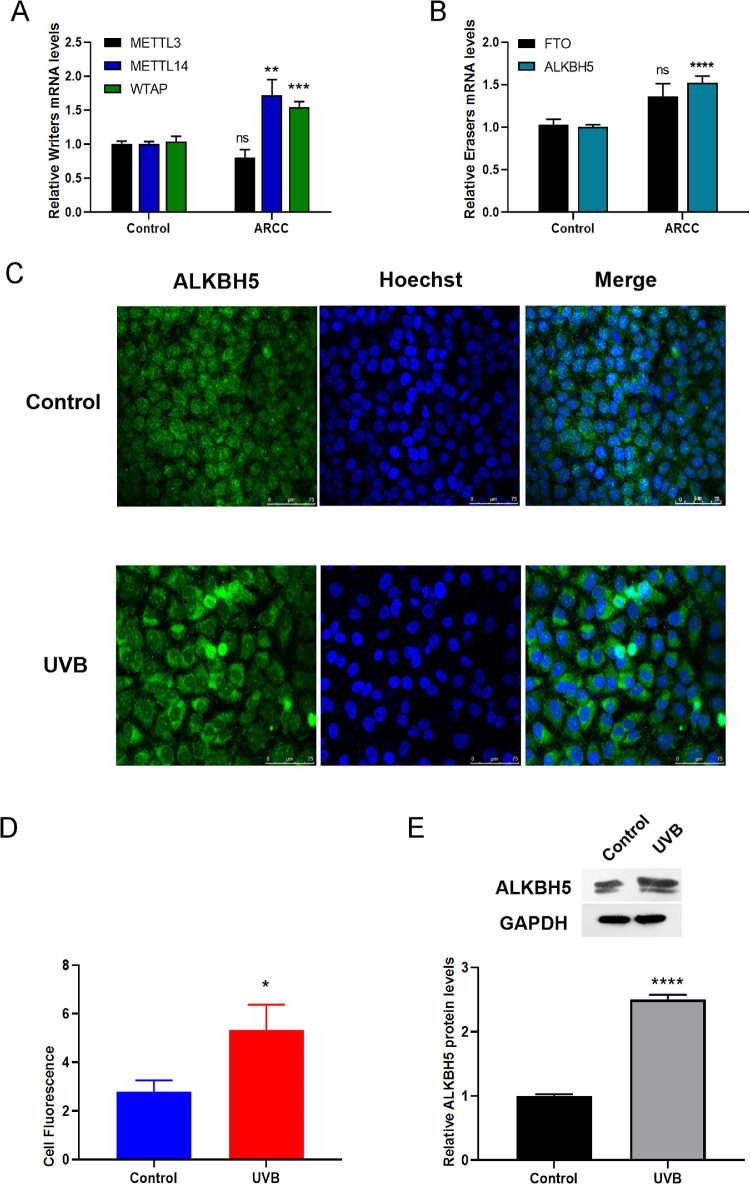
ALKBH5 was upregulated in ARC. (**A, B**) Quantitative real-time PCR was used to analysis mRNA level of *METTL3, METTL14, FTO, WTAP, ALKBH5* between in controls and ARCCs (n = 7 each). The data were normalized by level of GAPDH. (**C**) Representative ALKBH5/NeuN immunostaining between in control and UVB group. Scale bar: 75µm. (**D**) Quantification of the fluorescence intensity for ALKBH5. The ImageJ software was used to perform quantitative analysis. (**E**) The expression level of the ALKBH5 protein was significantly decreased in SRA01/04 after UVB exposure. ****P < 0.0001, ***P < 0.001, **P < 0.01, *P < 0.05, ns: no significance.

In ARCC groups, mRNA levels of ALKBH5 and METTL14, the key methyltransferases responsible for m^6^A modification, were significantly increased compared with the control groups (Figs. 6A, 6B). FTO and two major methyltransferases (METTL3 and WTAP) were not differentially regulated in ARCC groups.

Exposure of the lens to UVB induces DNA lesion and oxidative stress during the pathogenesis of ARC.^29^ Hence, the SRA01/04 cells were exposed to UVB light. Then, we found that ALKBH5 was primarily located in the cytoplasm and was upregulated in SRA01/04 cells after UVB irradiation by immunofluorescence (Figs. 6C, 6D). Furthermore, we validated the increased ALKBH5 protein level in the cells after radiation (Fig. 6E). Our data suggest that ALKBH5 may be largely responsible for the decreased m^6^A modification of circRNAs in ARC.

## Discussion

Many studies have shown the important role of epigenetic modifications in the pathogenesis of cataract.^7^^,^^30^^−^^32^ The expression profile and potential function of circRNAs in different tissues have been identified, including human lens tissues.^16^ Recently, m^6^A RNA modification has gained attention as a new epigenetic event. However, the role of this new RNA modification in epigenetics during ARC has not been characterized, especially in circRNA. As far as we know, the present study was the first to investigate the m^6^A-circRNA state in ARC by using RNA MeRIP-Seq. The results support the notion of a dynamic characteristic of m^6^A modification in LECs, which is associated with ARC pathogenesis.

We performed genome-wide profiling of m^6^A-tagged circRNAs between controls and ARCCs by using MeRIP-seq. In total, 2700 m^6^A peaks were significantly differentially expressed in circRNAs, with 1469 upregulated and 1231 downregulated. Our results demonstrated that the expression of differentially regulated circRNAs was comparable between controls and ARCCs, by RNA sequencing. There were 4233 upregulated circRNAs and 4561 downregulated circRNAs in ARCCs. Meanwhile, we compared the expression of m^6^A modified circRNAs and non-m^6^A modified circRNAs between in controls and ARCCs. In ARCC, m^6^A-levels in circRNAs are negatively correlated with the expression levels of circRNAs (*P* < 2.2e-16). The analyzed data showed that the expression of m^6^A-modified circRNAs in the ARCCs was lower than that of the controls, and the difference was statistically significant. What is more, these data underscore the dynamic characteristic between the m^6^A modifications at the circRNAs and expression of m^6^A circRNAs in ARCCs.

The discovery of m^6^A-circRNAs raises many questions that will need to be addressed in future work, including the significance of m^6^A-tagged on exons that compose circRNAs. A recent report showed that m^6^A-circRNAs are more commonly encoded by single exons and they tend to be longer than the exons of multiexon circRNAs for all groups of circRNAs in human embryonic stem cells (hESCs).^24^ Interestingly, we found that they also exist in LECs from ARCCs. Furthermore, it would be of interest to postulate whether exons methylated in mRNAs are the same exons that form m^6^A-circRNAs. Interestingly, m^6^A sites in mRNAs are most common in the last exon.^33^ However, the circularization of the last exon of genes are uncommon.^34^ Research suggested a different set of rules may govern m^6^A biogenesis in circRNAs compared with mRNAs.^24^ Further investigations would be needed to verify whether m^6^A-circRNAs exhibit distinct patterns of modifications compared with mRNAs in controls and ARCCs.

M^6^A modification is also implicated in the splicing of mRNAs, and they could be involved in alternative splicing of some circRNAs.^35^ Others reported that circRNA immunity has a considerable parallel to prokaryotic DNA restriction-modification system that transforms nucleic acid chemical modification into organismal innate immunity.^36^ Therefore GO and KEGG pathway analyses were performed to deduce potential functions of altered m^6^A modified transcripts. Afterward, conjoint analysis of MeRIP-seq and RNA-seq data identified m^6^A-circRNAs which were more hypomethylated and also significantly differentially expressed. Therefore it is necessary to further explore the function of m^6^A circRNAs.

M^6^A modification occurs via a methyltransferase complex (dedicated writers) mainly consisting of METTL3, METTL14, WTAP, and other components.^37^ This modification can be reversed by the demethylases (dedicated erasers) FTO and ALKBH5.^38^ Recently, studies reported that when the level of m^6^A mRNA modifications decreased, ALKBH5 were significantly upregulated in osteosarcoma (OS), lung adenocarcinoma and nucleus pulposus cells (NPCs).^39^^−^^41^ Similarly, ALKBH5 transcript levels were downregulated in clear cell renal cell carcinoma (ccRCC).^42^ Overall, these studies implicated ALKBH5 in playing a critical role in m^6^A modification. In our study, we found that ALKBH5 was upregulated in ARCCs. Then, we established a cataract oxidative damage model in cells by UVB irradiation. We also found the protein expression level of ALKBH5 was upregulated. Consistent with this result, total RNA m^6^A levels were increased in ARCCs.

M^6^A can be recognized by a number of m^6^A-binding proteins, which are responsible for exerting diverse effects of m^6^A on gene expression such as half-life,^43^ splicing,^44^ translational efficacy,^45^ nuclear export,^46^ and RNA structure.^44^ These effectors are called m^6^A “readers.” The majority of m^6^A readers are the YTH domain containing protein family, including YTHDF1, YTHDF2, YTHDF3, YTHDC1, and YTHDC2.^47^^,^^48^ Further investigations could help to verify the m^6^A-circRNA function between m^6^A-circRNAs and “readers” using RIP, RNA-pull-down, and qRT-PCR techniques.

A previous study reported that m^6^A modifications in circRNAs could be written and read by the same machinery (METTL3/14, YTH proteins) used for mRNAs but often at different locations.^24^ In general, m^6^A modifications play a role in mRNA stability and mediated by YTHDF2, but in contrast, a similar mechanism does not appear to promote degradation of circRNAs as it does for mRNAs. A recent study suggested a potential model wherein m^6^A-circRNAs and m^6^A-mRNAs encoded by the same exons are bundled together as part of a chromatin-associated liquid phase transition leading to a nuclear “liquid droplet”. Studies have also suggested that circRNAs, in general, may exhibit unique tuning qualities on liquid droplets.^43^^,^^49^ M^6^A-circRNAs may further modify these characteristics given their ability to interact with YTH proteins as well as other RNA binding proteins.^50^ Recently, there was also an article providing evidence to confirm the cross-talk between m^6^A modified mRNAs and circRNAs that affect mRNA half-life in a YTHDF2-dependent manner. However, in this study, it is unclear whether the recognition of m^6^A-circRNAs by “readers” plays a direct role.^24^ Taken together, we identified that circRNAs were more downregulated during increased m^6^A modification of circRNAs in our study.

Controlling the state of m^6^A modifications in circRNAs may act as a switch to control circRNA functionality. One of the key roles of circRNAs is to regulate gene expression.^51^ The regulatory mechanism of circRNAs has been a recent hotspot of research. Recently, an argument has been raised that only very limited circRNAs (maybe just several of them) could act as microRNA sponges.^52^ Lately, a special class of circRNAs as EIciRNAs (for example, circEIF3J and circPAIP2) was identified.^15^ In these circRNAs, exons are circularized with introns along with exons and are known as EIciRNAs. EIciRNAs might hold factors such as U1 snRNP through RNA-RNA interaction between U1 snRNA and EIciRNA, and then the EIciRNA–U1 snRNP complexes might further interact with the Pol II transcription complex at the promoters of parental genes to enhance gene expression. Once generated, EIciRNAs may modulate the expression of the parental genes transcriptionally to increase level of parental genes.^15^ Our previous studies found the involvements of aging, DNA damage, DNA repair, response to oxidative stress, proteolysis, ubiquitination, apoptosis, and autophagy in ARC reflecting a degree of similarity with ARC pathogenesis.^3^^−^^7^ The CircRNAs derived from these pathway genes may also play a regulatory role in the development of ARC.

As a novel epitranscriptomic marker, m^6^A is identified as a dynamic and reversible RNA modification in eukaryotes.^23^^,^^24^ Certainly, aberrant epigenetic patterns have already been linked to a number of age-related disorders, including cancer, Alzheimer's disease (AD), and autoimmune disorders.^53^^,^^54^ Thus ARC appears to be no exception.^55^ As mentioned above, circRNAs can regulate the expression of parental genes, thus affecting the biological functions of cells. The development of ARC is closely associated with many environmental risk factors, such as oxidative stress that could cause DNA damage.^7^ Thus it is possible to predict the function of some circRNAs via their host gene function. In ARC, oxidative stress is known to play an important role in disease pathogenesis.^7^ Hence, in the present study, combining analysis of differentially methylated circRNAs and differentially expressed circRNAs and the intersection of host genes associated with ARC pathogenesis, we enriched the circRNA of the host genes related to the mechanisms of ARC by GO analysis. We found that DNA damage repair and autophagy account for a large proportion of them. For studying these two mechanisms, we will conduct more in-depth research in the future.

## Conclusions

The level of m^6^A abundance in total circRNAs was decreased in the LECs from ARCCs in comparison with the controls. We also found that the expression of circRNAs was mostly decreased in the highly m^6^A-tagged circRNAs. The bioinformatics analysis predicted the potential functions of m^6^A modified circRNAs and the relevant pathways that may be associated with m^6^A modified circRNAs. Among five major methyltransferases, ALKBH5 was significantly upregulated in LECs of ARCCs. Our data provided novel evidence regarding the involvement of circRNAs m^6^A modifications in ARC. The altered expression of methyltransferases in lens tissue might selectively change the epigenetic profile of lens genome through regulating genes that host the circRNAs, thus enhance the susceptibility to ARC. The results might provide a new insight in the molecular target of ARC pathogenesis.

## Supplementary Material

Supplement 1
