# Supplementary material for: Identification and Characterization of N6-Methyladenosine CircRNAs and Methyltransferases in the Lens Epithelium Cells From Age-Related Cataract
Source: Invest Ophthalmol Vis Sci. 2020 Aug 6;61(10):13. doi: 10.1167/iovs.61.10.13 (PMC7441297; doi:10.1167/iovs.61.10.13)
Supplement: Supplement 1 [file iovs-61-10-13_s001.pdf]

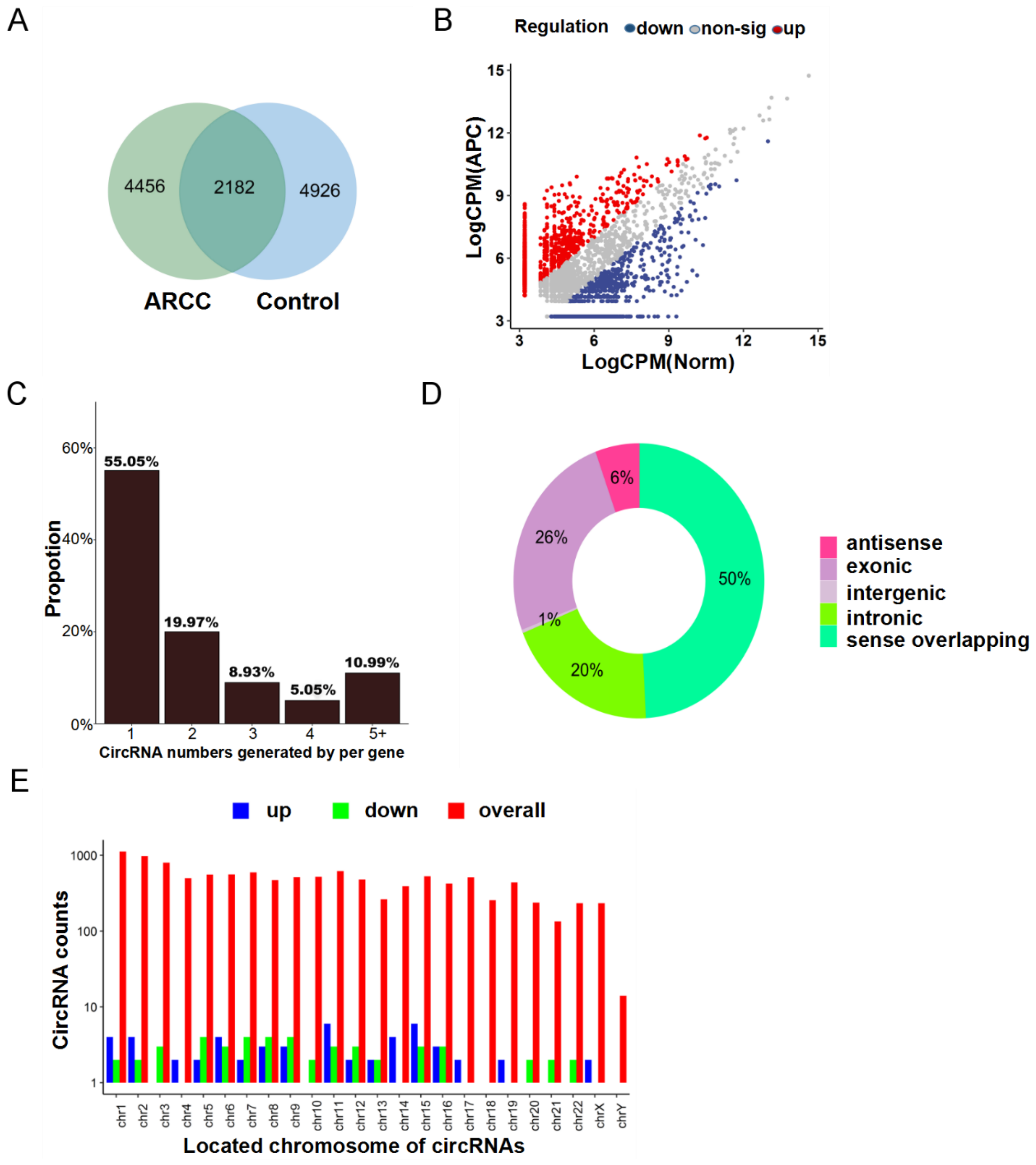

Figure S1. CircRNA profiling in control subjects versus ARCC patients. (A) Comparison of the expression circRNAs identified in control subjects and ARCC patients by Venn diagram. (B) Scatter plots showing the circRNAs that were differentially expressed between control subjects and ARCC patients with statistical significance (fold changes ≥1.5 and $p < 0.05$). (C) Proportion of circRNAs harboring different numbers by per genes. (D) Genomic distribution

of m^{6}A circRNAs. The percentage of m^{6}A-circRNAs identified under each condition is shown in parentheses. (E) Chromosomal distribution of the differentially expressed circRNAs.

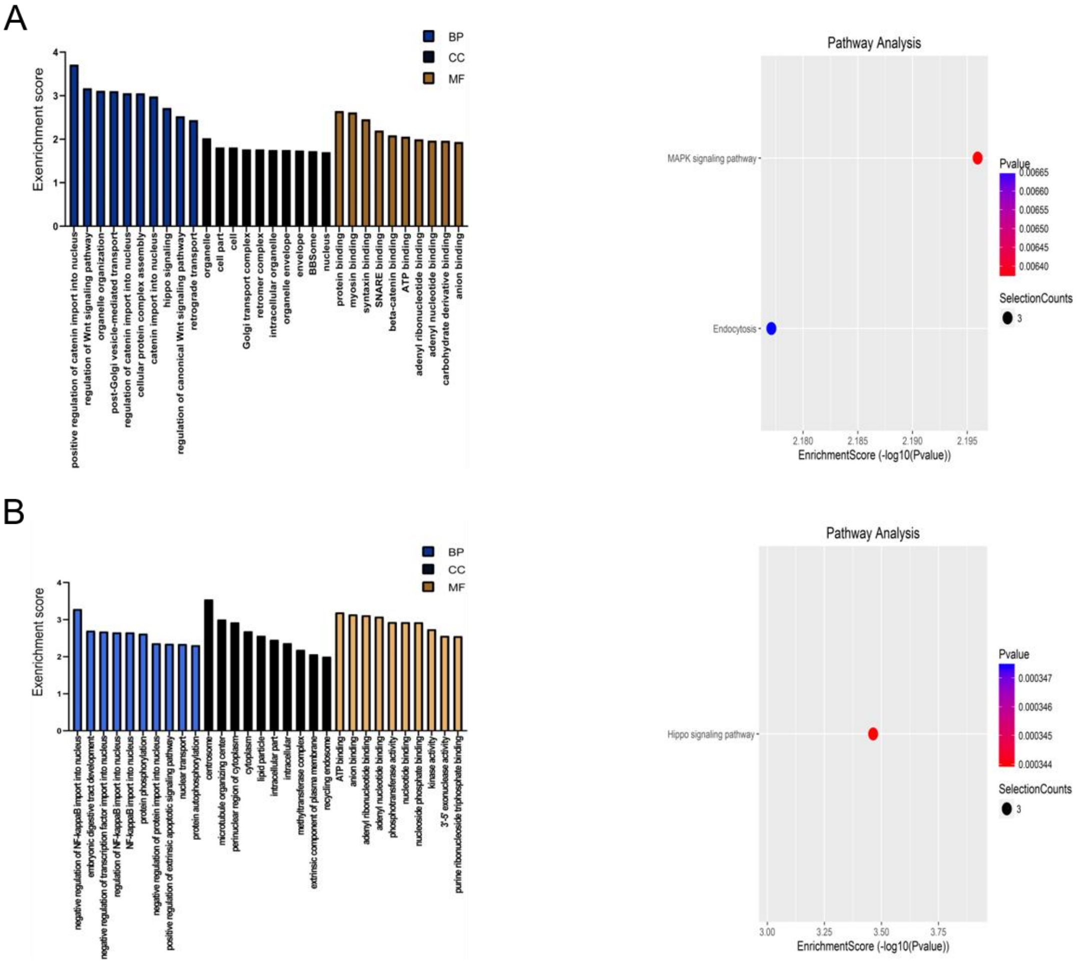

Figure S2. GO enrichment and KEGG signaling pathway analysis for host genes of upregulated (A) and downregulated (B) circRNAs. GO enrichment analysis include biological process (BP) analysis, cellular component (CC) analysis, and molecular function (MF) analysis. *P* values are calculated by DAVID tool.
